# (*Z*)-5-Benzyl­idene-3-butyl-4-phenyl-1,3-oxazolidin-2-one

**DOI:** 10.1107/S1600536811008762

**Published:** 2011-03-15

**Authors:** Jin-Wu Zhao, Jing-Xiu Xu

**Affiliations:** a School of Pharmacy, Guang Dong Medical College, Dong Guan 523808, People’s Republic of China

## Abstract

In the title compound, C_20_H_21_NO_2_, the benzyl group and the oxazolidin-2-one unit are each essentially planar, with maximum deviations of 0.026 (2) and 0.031 (2) Å, respectively. The dihedral angle between the phenyl ring and the oxazolidin-2-one unit is 69.25 (2)°. In the crystal, mol­ecules are linked by weak inter­molecular C—H⋯O and C—H⋯π inter­actions.

## Related literature

For general background to 2-oxazolidinone derivatives and for heterocyclic systems of anti­bacterial inter­est, see: Mukhtar & Wright (2005 ▶); Ager *et al.* (1996 ▶); Renslo *et al.* (2006 ▶). For bond-length data, see: Allen *et al.* (1987 ▶). For the chemical structure of the title compound established from NMR data, see: Yoo & Li (2008 ▶).
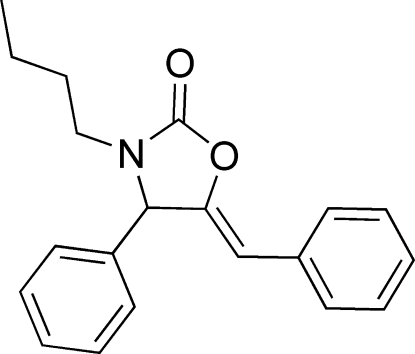


## Experimental

### 

#### Crystal data

C_20_H_21_NO_2_
*M* *_r_* = 307.38
Monoclinic, 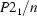

*a* = 10.029 (2) Å
*b* = 9.1941 (18) Å
*c* = 18.389 (4) Å
β = 100.51 (3)°
*V* = 1667.1 (6) Å^3^
*Z* = 4
Mo *K*α radiation
μ = 0.08 mm^−1^
*T* = 293 K
0.31 × 0.25 × 0.18 mm

#### Data collection

Rigaku/MSC Mercury CCD diffractometer
Absorption correction: multi-scan (*REQAB*; Jacobson, 1998 ▶) *T*_min_ = 0.989, *T*_max_ = 0.997
12969 measured reflections
2994 independent reflections
1803 reflections with *I* > 2σ(*I*)
*R*_int_ = 0.041

#### Refinement

*R*[*F* ^2^ > 2σ(*F* ^2^)] = 0.042
*wR*(*F* ^2^) = 0.117
*S* = 1.03
2994 reflections
209 parameters
H-atom parameters constrained
Δρ_max_ = 0.17 e Å^−3^
Δρ_min_ = −0.18 e Å^−3^

### 

Data collection: *RAPID-AUTO* (Rigaku, 1998 ▶); cell refinement: *RAPID-AUTO*; data reduction: *CrystalStructure* (Rigaku/MSC, 2002 ▶); program(s) used to solve structure: *SHELXS97* (Sheldrick, 2008 ▶); program(s) used to refine structure: *SHELXL97* (Sheldrick, 2008 ▶); molecular graphics: *ORTEPII* (Johnson, 1976 ▶); software used to prepare material for publication: *SHELXL97*.

## Supplementary Material

Crystal structure: contains datablocks I, global. DOI: 10.1107/S1600536811008762/bg2393sup1.cif

Structure factors: contains datablocks I. DOI: 10.1107/S1600536811008762/bg2393Isup2.hkl

Additional supplementary materials:  crystallographic information; 3D view; checkCIF report

## Figures and Tables

**Table 1:** Hydrogen-bond geometry (Å, °) *Cg*1 and *Cg*2 are the centroids of the C1–C6 and C11–C16 rings, respectively.

*D*—H⋯*A*	*D*—H	H⋯*A*	*D*⋯*A*	*D*—H⋯*A*
C4—H4⋯O2^i^	0.93	2.58	3.343 (3)	141
C3—H3⋯*Cg*2^ii^	0.93	2.93	3.674 (2)	138
C12—H12⋯*Cg*1^iii^	0.93	2.91	3.706 (3)	145
C20—H20*B*⋯*Cg*2^iv^	0.96	2.92	3.812 (1)	156

Symmetry codes: (i) 
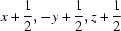
; (ii) 
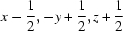
; (iii) 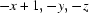; (iv) 
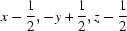
.

**Fig. 1.**
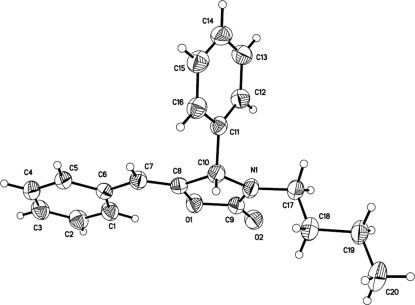
The molecular structure of the tile compound, showing 50% probability displacement ellipsoids and the atom-numbering scheme.

**Fig. 2.**
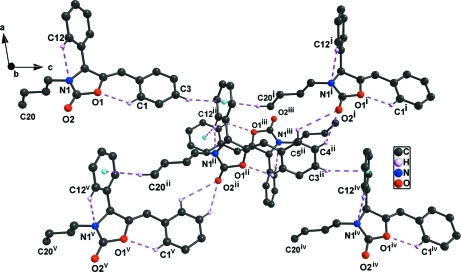
Packing diagram of the title compound; C—H···O, C—H···N and C—H···π interactions are shown as dashed lines. The H-atoms not involved in H-bonds have been excluded for clarity.

## supplementary crystallographic information

### Comment

The title compound, (I), C_19_H_17_N_3_O_2_, is a 2-oxazolidinone derivative. The 2-oxazolidinone ring is an important heterocyclic structural unit. It possesses significant antibacterial activities and plays an important role as an intermediate for the synthesis of more complex active organic compounds and further functionalized heterocyclic systems of antibacterial interest (Mukhtar *et al.*, 2005; Ager *et al.*, 1996; Renslo *et al.*, 2006). The structure of title compound has been established from the NMR data (Yoo *et al.*, 2008). However, the crystal structure of title compound has not been reported. In view of this, the crystal structure determination of the title compound was carried out and the results are presented here.

As depicted in Fig. 1, the benzyl group A (C1-C7)  and the heterocyclic ring of 2-oxazolidinone B (N1/C8-C10/O1/O2)  are almost planar with maximum deviations of 0.024 (2) Å for C7 and 0.031 (2) Å for O2, respectively, and determine a dihedral angle of 2.56 (2) °. The phenyl ring C (C11—C16) is of also planar (max. deviation 0.007 (2) Å for C15).

The butyl moiety (C17—C20) adopts a slight twist conformation with C20 displaced by 0.123 (3) Å from the plane defined by the atoms C17—C19 (D). The dihedral angles between C/A, C/B, C/D, D/A and D/B are 69.05 (2) °, 69.30 (3) °, 75.17 (1) °, 85.79 (4) ° and 83.29 (2) °, respectively. The bond lengths and bond angles are within normal range (Allen *et al.*, 1987). The molecules are linked into a three-dimensional supramolecular network through intermolecular C—H···O hydrogen bonding interactions and C—H···π stacking interactions (Table 1, Fig. 2). The H-to-centroid distances of H3···*Cg*2^i^ = 2.93 (2), H12···*Cg*1^ii^ = 2.91 (3) and H20B···*Cg*2^iii^ = 2.91 (4) [*Cg*1 and *Cg*2 are the centroids of the C1, C2, C3, C4, C5, C6 ring, and C11, C12, C13, C14, C15, C16 ring, respectively. Symmetry codes: (i)-1/2 + *x*, 1/2 - *y*, 1/2 + *z*; (ii)3/2 - *x*, 1/2 + *y*, 1/2 - *z*; (iii)-1/2 + *x*, 1/2 - *y*, -1/2 + *z*]. In addition, intramolecular C—H···O and C—H···N hydrogen bonds are also observed.

### Experimental

A 15 ml polytetrafluoroethylene (PTFE) reaction vessel was charged with copper(I)iodide (0.6 mmol, 0.114 g), butylamine (4 mmol, 0.293 g), benzaldehyde (4 mmol, 0.425 g) and ethynylbenzene (3 mmol, 0.204 g). Then the vessel was fixed into a stainless steel autoclave with a pressure-regulating system. The autoclave was sealed. Liquid CO_2_ was introduced from a cylinder and the reaction mixture was magnetically stirred at 373 K under 8 MPa for 12 h. The vessel was cooled with an ice bath and the pressure was released slowly to atmospheric pressure after the reaction completed. The reaction mixture was flushed with EtOAc (30 ml) and the ethyl acetate fractions were combined. The resulting solvent was placed through a plug of silica gel, and then evaporated. The residue was purified by silica gel (200–300 mesh) column by elution with MSO:EtOAc (8:1) to give 20 fractions (200 ml per fraction). The title compound (539.6 mg) was isolated from the fractions 3–17 (yield 71.2%). Single crystals suitable for X-ray deffraction were prepared by slov evaporation of a solution of the title compound in petroleum ether at room temperature.

### Refinement

All H atoms were located on the difference maps, and were treated as riding atoms with C—H distances of 0.93, 0.96, 0.97 and 0.98 Å, for aryl, methyl, methine and tertiary alkyl, respectively, with *U*_iso_(H) = 1.5*U*_eq_ (methyl C-atoms) and 1.2*U*_eq_(non-methyl C-atoms). The hightest peak is located 1.54 Å from C1 and the deepest hole is located 0.86 Å from H13.

### Crystal data

**Table:**

C_20_H_21_NO_2_	*F*(000) = 656
*M**_r_* = 307.38	*D*_x_ = 1.225 Mg m^−^^3^
Monoclinic, *P*2_1_/*n*	Mo *K*α radiation, λ = 0.71073 Å
Hall symbol: -P 2yn	Cell parameters from 5837 reflections
*a* = 10.029 (2) Å	θ = 2.8–27.9°
*b* = 9.1941 (18) Å	µ = 0.08 mm^−^^1^
*c* = 18.389 (4) Å	*T* = 293 K
β = 100.51 (3)°	Block, colorless
*V* = 1667.1 (6) Å^3^	0.31 × 0.25 × 0.18 mm
*Z* = 4	

### Data collection

**Table:**

Rigaku/MSC Mercury CCD diffractometer	2994 independent reflections
Radiation source: fine-focus sealed tube	1803 reflections with *I* > 2σ(*I*)
graphite	*R*_int_ = 0.041
ω scans	θ_max_ = 25.2°, θ_min_ = 3.0°
Absorption correction: multi-scan (REQAB; Jacobson, 1998)	*h* = −12→12
*T*_min_ = 0.989, *T*_max_ = 0.997	*k* = −11→11
12969 measured reflections	*l* = −22→21

### Refinement

**Table:**

Refinement on *F*^2^	Primary atom site location: structure-invariant direct methods
Least-squares matrix: full	Secondary atom site location: difference Fourier map
*R*[*F*^2^ > 2σ(*F*^2^)] = 0.042	Hydrogen site location: inferred from neighbouring sites
*wR*(*F*^2^) = 0.117	H-atom parameters constrained
*S* = 1.03	*w* = 1/[σ^2^(*F*_o_^2^) + (0.0472*P*)^2^ + 0.3223*P*] where *P* = (*F*_o_^2^ + 2*F*_c_^2^)/3
2994 reflections	(Δ/σ)_max_ < 0.001
209 parameters	Δρ_max_ = 0.17 e Å^−^^3^
0 restraints	Δρ_min_ = −0.18 e Å^−^^3^

### Special details

**Table:**

Geometry. All e.s.d.'s (except the e.s.d. in the dihedral angle between two l.s. planes) are estimated using the full covariance matrix. The cell e.s.d.'s are taken into account individually in the estimation of e.s.d.'s in distances, angles and torsion angles; correlations between e.s.d.'s in cell parameters are only used when they are defined by crystal symmetry. An approximate (isotropic) treatment of cell e.s.d.'s is used for estimating e.s.d.'s involving l.s. planes.
Refinement. Refinement of *F*^2^ against ALL reflections. The weighted *R*-factor *wR* and goodness of fit *S* are based on *F*^2^, conventional *R*-factors *R* are based on *F*, with *F* set to zero for negative *F*^2^. The threshold expression of *F*^2^ > σ(*F*^2^) is used only for calculating *R*-factors(gt) *etc*. and is not relevant to the choice of reflections for refinement. *R*-factors based on *F*^2^ are statistically about twice as large as those based on *F*, and *R*- factors based on ALL data will be even larger.

### Fractional atomic coordinates and isotropic or equivalent isotropic displacement parameters (Å^2^)

**Table:**

	*x*	*y*	*z*	*U*_iso_*/*U*_eq_	
C1	0.59314 (19)	0.3280 (2)	0.36032 (11)	0.0590 (5)	
H1	0.5441	0.3603	0.3154	0.071*	
C2	0.5426 (2)	0.3511 (3)	0.42412 (12)	0.0675 (6)	
H2	0.4609	0.4003	0.4218	0.081*	
C3	0.6115 (2)	0.3023 (3)	0.49109 (12)	0.0680 (6)	
H3	0.5764	0.3174	0.5339	0.082*	
C4	0.7330 (2)	0.2308 (2)	0.49425 (11)	0.0636 (6)	
H4	0.7798	0.1966	0.5393	0.076*	
C5	0.78512 (19)	0.2099 (2)	0.43106 (10)	0.0532 (5)	
H5	0.8682	0.1630	0.4342	0.064*	
C6	0.71618 (17)	0.2574 (2)	0.36199 (10)	0.0477 (5)	
C7	0.77658 (18)	0.2274 (2)	0.29672 (10)	0.0517 (5)	
H7	0.8565	0.1737	0.3059	0.062*	
C8	0.73519 (17)	0.2650 (2)	0.22699 (10)	0.0477 (5)	
C9	0.6003 (2)	0.3656 (2)	0.12780 (11)	0.0560 (5)	
C10	0.79952 (18)	0.2321 (2)	0.16033 (10)	0.0509 (5)	
H10	0.7967	0.1270	0.1512	0.061*	
C11	0.94436 (17)	0.2858 (2)	0.16712 (9)	0.0471 (5)	
C12	0.9744 (2)	0.4219 (2)	0.14300 (11)	0.0569 (5)	
H12	0.9047	0.4837	0.1217	0.068*	
C13	1.1078 (2)	0.4670 (3)	0.15040 (12)	0.0669 (6)	
H13	1.1271	0.5584	0.1333	0.080*	
C14	1.2119 (2)	0.3782 (3)	0.18269 (12)	0.0688 (6)	
H14	1.3013	0.4090	0.1874	0.083*	
C15	1.1831 (2)	0.2442 (3)	0.20790 (12)	0.0678 (6)	
H15	1.2532	0.1842	0.2306	0.081*	
C16	1.05059 (19)	0.1974 (2)	0.19989 (11)	0.0585 (5)	
H16	1.0322	0.1054	0.2167	0.070*	
C17	0.7052 (2)	0.2948 (3)	0.02324 (10)	0.0651 (6)	
H17A	0.7983	0.3011	0.0159	0.078*	
H17B	0.6563	0.3771	−0.0016	0.078*	
C18	0.6430 (2)	0.1560 (2)	−0.01191 (10)	0.0643 (6)	
H18A	0.5527	0.1447	−0.0008	0.077*	
H18B	0.6971	0.0738	0.0094	0.077*	
C19	0.6343 (2)	0.1550 (3)	−0.09517 (10)	0.0707 (6)	
H19A	0.7254	0.1578	−0.1060	0.085*	
H19B	0.5875	0.2421	−0.1158	0.085*	
C20	0.5609 (3)	0.0224 (3)	−0.13268 (12)	0.0880 (8)	
H20A	0.6069	−0.0643	−0.1128	0.132*	
H20B	0.5600	0.0270	−0.1849	0.132*	
H20C	0.4694	0.0210	−0.1240	0.132*	
N1	0.70329 (15)	0.30487 (19)	0.10237 (8)	0.0570 (5)	
O1	0.61860 (12)	0.34597 (15)	0.20363 (7)	0.0572 (4)	
O2	0.50276 (13)	0.43149 (18)	0.09551 (8)	0.0707 (5)	

### Atomic displacement parameters (Å^2^)

**Table:**

	*U*^11^	*U*^22^	*U*^33^	*U*^12^	*U*^13^	*U*^23^
C1	0.0542 (12)	0.0656 (14)	0.0581 (12)	0.0036 (10)	0.0127 (10)	0.0080 (10)
C2	0.0576 (12)	0.0786 (16)	0.0709 (14)	0.0087 (12)	0.0242 (11)	0.0050 (12)
C3	0.0719 (14)	0.0809 (17)	0.0569 (13)	−0.0049 (13)	0.0272 (11)	−0.0012 (11)
C4	0.0655 (13)	0.0775 (16)	0.0483 (12)	−0.0063 (12)	0.0121 (10)	0.0030 (10)
C5	0.0486 (10)	0.0606 (13)	0.0501 (11)	−0.0024 (9)	0.0079 (9)	0.0028 (9)
C6	0.0439 (10)	0.0522 (12)	0.0478 (11)	−0.0035 (9)	0.0103 (8)	0.0021 (9)
C7	0.0475 (10)	0.0575 (13)	0.0496 (11)	0.0031 (9)	0.0079 (9)	0.0033 (9)
C8	0.0403 (9)	0.0536 (12)	0.0484 (11)	0.0000 (9)	0.0061 (8)	0.0021 (9)
C9	0.0478 (11)	0.0695 (14)	0.0498 (11)	−0.0064 (11)	0.0066 (9)	0.0065 (10)
C10	0.0509 (10)	0.0564 (12)	0.0440 (10)	0.0004 (9)	0.0050 (8)	−0.0012 (9)
C11	0.0451 (10)	0.0569 (13)	0.0397 (10)	0.0054 (9)	0.0090 (8)	−0.0048 (9)
C12	0.0545 (12)	0.0586 (14)	0.0572 (12)	0.0060 (10)	0.0087 (9)	0.0024 (10)
C13	0.0655 (13)	0.0702 (15)	0.0667 (14)	−0.0091 (12)	0.0163 (11)	−0.0021 (11)
C14	0.0495 (12)	0.0943 (19)	0.0639 (13)	−0.0051 (13)	0.0134 (10)	−0.0137 (13)
C15	0.0531 (12)	0.0852 (18)	0.0638 (14)	0.0157 (12)	0.0074 (10)	−0.0040 (12)
C16	0.0573 (12)	0.0622 (14)	0.0551 (12)	0.0092 (11)	0.0074 (10)	0.0002 (10)
C17	0.0596 (12)	0.0931 (17)	0.0412 (11)	−0.0056 (12)	0.0060 (9)	0.0018 (11)
C18	0.0695 (13)	0.0788 (16)	0.0429 (11)	0.0114 (12)	0.0061 (9)	0.0005 (10)
C19	0.0640 (13)	0.1041 (19)	0.0441 (11)	0.0091 (13)	0.0105 (10)	−0.0017 (12)
C20	0.122 (2)	0.091 (2)	0.0481 (13)	0.0186 (16)	0.0078 (13)	−0.0100 (12)
N1	0.0505 (9)	0.0798 (13)	0.0386 (9)	0.0037 (9)	0.0026 (7)	0.0022 (8)
O1	0.0494 (8)	0.0730 (10)	0.0493 (8)	0.0095 (7)	0.0088 (6)	0.0084 (7)
O2	0.0487 (8)	0.0929 (12)	0.0676 (9)	0.0062 (8)	0.0029 (7)	0.0216 (8)

### Geometric parameters (Å, °)

**Table:**

C1—C2	1.376 (3)	C11—C16	1.387 (3)
C1—C6	1.390 (3)	C12—C13	1.383 (3)
C1—H1	0.9300	C12—H12	0.9300
C2—C3	1.373 (3)	C13—C14	1.372 (3)
C2—H2	0.9300	C13—H13	0.9300
C3—C4	1.376 (3)	C14—C15	1.365 (3)
C3—H3	0.9300	C14—H14	0.9300
C4—C5	1.372 (3)	C15—C16	1.379 (3)
C4—H4	0.9300	C15—H15	0.9300
C5—C6	1.401 (2)	C16—H16	0.9300
C5—H5	0.9300	C17—N1	1.461 (2)
C6—C7	1.466 (3)	C17—C18	1.513 (3)
C7—C8	1.320 (2)	C17—H17A	0.9700
C7—H7	0.9300	C17—H17B	0.9700
C8—O1	1.387 (2)	C18—C19	1.518 (3)
C8—C10	1.516 (3)	C18—H18A	0.9700
C9—O2	1.210 (2)	C18—H18B	0.9700
C9—N1	1.332 (2)	C19—C20	1.523 (3)
C9—O1	1.385 (2)	C19—H19A	0.9700
C10—N1	1.463 (2)	C19—H19B	0.9700
C10—C11	1.518 (2)	C20—H20A	0.9600
C10—H10	0.9800	C20—H20B	0.9600
C11—C12	1.380 (3)	C20—H20C	0.9600
			
C2—C1—C6	121.09 (19)	C14—C13—H13	119.7
C2—C1—H1	119.5	C12—C13—H13	119.7
C6—C1—H1	119.5	C15—C14—C13	119.5 (2)
C1—C2—C3	120.7 (2)	C15—C14—H14	120.2
C1—C2—H2	119.6	C13—C14—H14	120.2
C3—C2—H2	119.6	C14—C15—C16	120.3 (2)
C4—C3—C2	119.4 (2)	C14—C15—H15	119.9
C4—C3—H3	120.3	C16—C15—H15	119.9
C2—C3—H3	120.3	C15—C16—C11	120.8 (2)
C5—C4—C3	120.2 (2)	C15—C16—H16	119.6
C5—C4—H4	119.9	C11—C16—H16	119.6
C3—C4—H4	119.9	N1—C17—C18	113.57 (17)
C4—C5—C6	121.53 (19)	N1—C17—H17A	108.9
C4—C5—H5	119.2	C18—C17—H17A	108.9
C6—C5—H5	119.2	N1—C17—H17B	108.9
C1—C6—C5	117.08 (18)	C18—C17—H17B	108.9
C1—C6—C7	124.57 (17)	H17A—C17—H17B	107.7
C5—C6—C7	118.34 (17)	C17—C18—C19	112.31 (18)
C8—C7—C6	130.01 (18)	C17—C18—H18A	109.1
C8—C7—H7	115.0	C19—C18—H18A	109.1
C6—C7—H7	115.0	C17—C18—H18B	109.1
C7—C8—O1	122.65 (17)	C19—C18—H18B	109.1
C7—C8—C10	128.95 (17)	H18A—C18—H18B	107.9
O1—C8—C10	108.40 (15)	C18—C19—C20	113.3 (2)
O2—C9—N1	130.30 (19)	C18—C19—H19A	108.9
O2—C9—O1	120.42 (19)	C20—C19—H19A	108.9
N1—C9—O1	109.27 (17)	C18—C19—H19B	108.9
N1—C10—C8	100.16 (15)	C20—C19—H19B	108.9
N1—C10—C11	113.93 (16)	H19A—C19—H19B	107.7
C8—C10—C11	114.25 (15)	C19—C20—H20A	109.5
N1—C10—H10	109.4	C19—C20—H20B	109.5
C8—C10—H10	109.4	H20A—C20—H20B	109.5
C11—C10—H10	109.4	C19—C20—H20C	109.5
C12—C11—C16	118.39 (18)	H20A—C20—H20C	109.5
C12—C11—C10	122.06 (17)	H20B—C20—H20C	109.5
C16—C11—C10	119.54 (19)	C9—N1—C10	112.76 (15)
C13—C12—C11	120.3 (2)	C9—N1—C17	121.86 (16)
C13—C12—H12	119.8	C10—N1—C17	124.73 (16)
C11—C12—H12	119.8	C9—O1—C8	109.36 (15)
C14—C13—C12	120.6 (2)		

### Hydrogen-bond geometry (Å, °)

**Table:**

Cg1 and Cg2 are the centroids of the C1–C6 and C11–C16 rings, respectively.

**Table:**

*D*—H···*A*	*D*—H	H···*A*	*D*···*A*	*D*—H···*A*
C4—H4···O2^i^	0.93	2.58	3.343 (3)	141
C3—H3···Cg2^ii^	0.93	2.93	3.674 (2)	138
C12—H12···Cg1^iii^	0.93	2.91	3.706 (3)	145
C20—H20B···Cg2^iv^	0.96	2.92	3.812 (1)	156

Symmetry codes: (i) *x*+1/2, −*y*+1/2, *z*+1/2; (ii) *x*−1/2, −*y*+1/2, *z*+1/2; (iii) −*x*+1, −*y*, −*z*; (iv) *x*−1/2, −*y*+1/2, *z*−1/2.

## References

[bb1] Ager, D. J., Prakash, I. & Schaad, D. R. (1996). *Chem. Rev.* **96**, 835–876. 10.1021/cr9500038 11848773

[bb2] Allen, F. H., Kennard, O., Watson, D. G., Brammer, L., Orpen, A. G. & Taylor, R. (1987). *J. Chem. Soc. Perkin Trans. 2*, pp. S1–19.

[bb3] Jacobson, R. (1998). *REQAB* Molecular Structure Corporation, The Woodlands, Texas, USA.

[bb4] Johnson, C. K. (1976). *ORTEPII* Report ORNL-5138. Oak Ridge National Laboratory, Tennessee, USA.

[bb5] Mukhtar, T. A. & Wright, G. D. (2005). *Chem. Rev.* **105**, 529–452. 10.1021/cr030110z 15700955

[bb6] Renslo, A. R., Luehr, G. W. & Gordeev, M. F. (2006). *Bioorg. Med. Chem.* **14**, 4227–4240. 10.1016/j.bmc.2006.01.068 16527486

[bb7] Rigaku (1998). *RAPID-AUTO* Rigaku Corporation, Tokyo, Japan.

[bb8] Rigaku/MSC (2002). *CrystalStructure* Rigaku/MSC, The Woodlands, Texas, USA.

[bb9] Sheldrick, G. M. (2008). *Acta Cryst.* A**64**, 112–122. 10.1107/S0108767307043930 18156677

[bb10] Yoo, W. J. & Li, C. J. (2008). *Adv. Synth. Catal.* **350**, 1503–1506.

